# Commentary: Preservation of a remote fear memory requires new myelin formation

**DOI:** 10.3389/fncir.2020.00028

**Published:** 2020-05-19

**Authors:** Yini Li, Haibo Shi

**Affiliations:** ^1^Department of Otorhinolaryngology-Head and Neck Surgery, The Sixth People's Hospital affiliated to Shanghai Jiao Tong University, Shanghai, China; ^2^Shanghai Key Laboratory of Sleep Disordered Breathing, Shanghai, China

**Keywords:** memory, oligodendrogenesis, myelination, reconsolidation, experience

Memory is not permanently fixed, but can be updated by experience continuously. This process requires synaptic reorganization of neural circuits, such as synaptic plasticity. In addition, adult oligodendrogenesis throughout lifetime provides a plastic and perseverant anatomical substrate for shaping the functioning for these neural circuits (Tripathi et al., 2017; Doron and Goshen, 2020). Accumulating evidence demonstrated that oligodendrogenesis is regulated by neuronal activity and plays important roles in experience-dependent learning, such as through *de novo* myelination with temporal precision (Gibson et al., 2014; McKenzie et al., 2014; Mensch et al., 2015; Xiao et al., 2016). Recently, several studies highlighted the roles of new myelin formation in memory processing, especially in reconsolidation of remote memory (Pan et al., 2020; Steadman et al., 2020).

Steadman et al. (2020) reported that the experience-driven oligodendrogenesis is crucial for spatial memory acquisition and consolidation. The study confirmed the oligodendrogenesis induced by memory formation and investigated their causal relationship with a spatial memory task in water maze. The animals took tests 1 day and 1 month following training to separate the processes of memory acquisition and consolidation, respectively. To confirm the involvement of oligodendrogenesis, they genetically controlled adult oligodendrogenesis at different stages. Results showed that reduced adult oligodendrogenesis during immediate but not later post-training period impaired both spatial learning and memory consolidation, suggesting a temporal control for efficient myelination as an adaptive response to neural circuit activities. With a contextual fear conditioning paradigm, the authors further reported the potential importance of hippocampal-cortical ripple-spindle coupling, which correlated to oligodendrogenesis.

Consistently, Pan et al. (2020) demonstrated a similar role of oligodendrogenesis and myelination with a prolonged timescale (up to several weeks). They trained animals for fear learning in a contextual fear memory task and focused on the medial prefrontal cortex (mPFC). Notably, context-elicited freezing responses of transgenic mice with new myelin formation eliminated exhibited no difference during recent retrieval sessions (24 h) compared to normal controls, but declined at 30 days post-conditioning (remote). In addition, they recorded population calcium dynamics with fiber photometry in the mPFC and detected a temporal pattern of prefrontal activity, which decreased at 24 h post-conditioning and elevated at 30 days post-conditioning. However, mice lacking active oligodendrogenesis failed to exhibit such time-dependent changes. With pharmacological agents facilitating new myelin formation (e.g., clemastine fumarate), they further confirmed the dependence of oligodendrogenesis and myelination for stabilizing remote fear memory and promoting fear generalization.

Both studies demonstrated the importance of adult oligodendrogenesis and new myelin formation for remote memory retrieval. These findings accord well with previous studies in motor learning and sensory enrichment, which induce oligodendrogenesis in motor and somatosensory cortices, respectively (McKenzie et al., 2014; Xiao et al., 2016; Hughes et al., 2018). With electron microscopy, Steadman et al. (2020) excluded the influence of myelin thickness by measuring the g-ratio of myelinated axons; while Pan et al. (2020) showed a significant increase in the density of myelinated axons, both consistent with experience-driven *de novo* myelination in adult mice reported previously (Hill et al., 2018; Hughes et al., 2018). In addition, both studies utilized a temporally controlled strategy to figure out the time frame in which oligodendrogenesis involved in different stages of memory processing; they demonstrated the value of oligodendrogenesis during a small time window to consolidation period. To further explore the underlying mechanism, both studies tried to explain the interaction between new myelinating oligodendrocytes and neurons through electrophysiological or calcium imaging method. Together with previous studies (Adamsky et al., 2018; Alberini et al., 2018; Koeppen et al., 2018), all these evidences emphasize a brand-new understanding of how non-neuronal cells shape neural circuits, thus modulating cognitive processes.

Yet, there are some issues requiring further exploration. For instance, Pan et al. (2020) reported that there was hardly new myelin formation until at least 7 days later though maturing into pre-myelinating oligodendrocyte could be fast. While in previous study using a skill learning model (Xiao et al., 2016), the behavioral performance of two genotype groups diverged within first 12 h. This may indicate different neural mechanisms underlying different types of learning and memory acquisition (Fields and Bukalo, 2020), and an extra role of oligodendrocyte precursors or pre-myelinating oligodendrocytes beyond myelination. As for fear memory such as post-traumatic stress disorders (PTSD), time scale is especially important, since its treatment strategy mainly targets reconsolidation and extinction following retrieval (Kida, 2019). Steadman et al. (2020) emphasized that the impairment of memory consolidation was dependent on the time to block oligodendrogenesis. Optimizing a precise time window of myelination may offer a better fundamentally-reorganized neural circuit for the forgetting of traumatic memories.

Fear memories are reported to depend on coordinated activity across interconnected brain regions, especially including hippocampus, mPFC, and amygdala (Herry and Johansen, 2014). Precise spike-time arrival determined by myelination could be critical in such oscillation couplings. Steadman et al. (2020) proposed coupling between hippocampal sharp wave ripples (SWR) and cortical spindles promoted by new myelination pattern as the regulator for memory consolidation, which is supported by a gain-of-function study of hippocampal–cortical coordination during the SWR (Maingret et al., 2016). As a pattern associated with highly synchronous neural firing in the hippocampus and modulation of neural activity in distributed brain regions, SWR reaches apex rate in the contexts of novelty and reward (Joo and Frank, 2018), consistent with functions in both memory consolidation and retrieval, thus could be a promising direction for future studies. In line with improved remote memory preservation after chronic pro-myelinating agent administration reported by Pan et al. (2020) and Wang et al. (2020) demonstrated both genetically and pharmacologically enhancing myelination can rescue spatial memory decline during aging, implying therapeutic potential of targeting myelination regulation in memory issues.

In conclusion, adult oligodendrogenesis is essential in shaping memory circuits by promoting myelination, thus influence the acquisition, retrieval, consolidation, and reconsolidation at different stages of memory. Targeting myelination regulation might serve as a new approach for memory enhancement and restoration.

## Author Contributions

All authors designed the study together and wrote the manuscript together.

## Conflict of Interest

The authors declare that the research was conducted in the absence of any commercial or financial relationships that could be construed as a potential conflict of interest.
